# Supplementary material for: Deep mutation, insertion and deletion scanning across the Enterovirus A proteome reveals constraints shaping viral evolution
Source: Nat Microbiol. 2024 Nov 28;10(1):158–68. doi: 10.1038/s41564-024-01871-y (PMC11726453; doi:10.1038/s41564-024-01871-y)
Supplement: Supplementary file 1 — Supplementary Figs. 1–3. [file 41564_2024_1871_MOESM1_ESM.pdf]

nature microbiology

Supplementary information https://doi.org/10.1038/s41564-024-01871-y

Deep mutation, insertion and deletion scanning across the Enterovirus A proteome reveals constraints shaping viral evolution

In the format provided by the authors and unedited

EV-A71 Deep Mutation, Insertion, and Deletion Scanning
Bakhache, et al.
Nature Microbiology
Supplementary Information

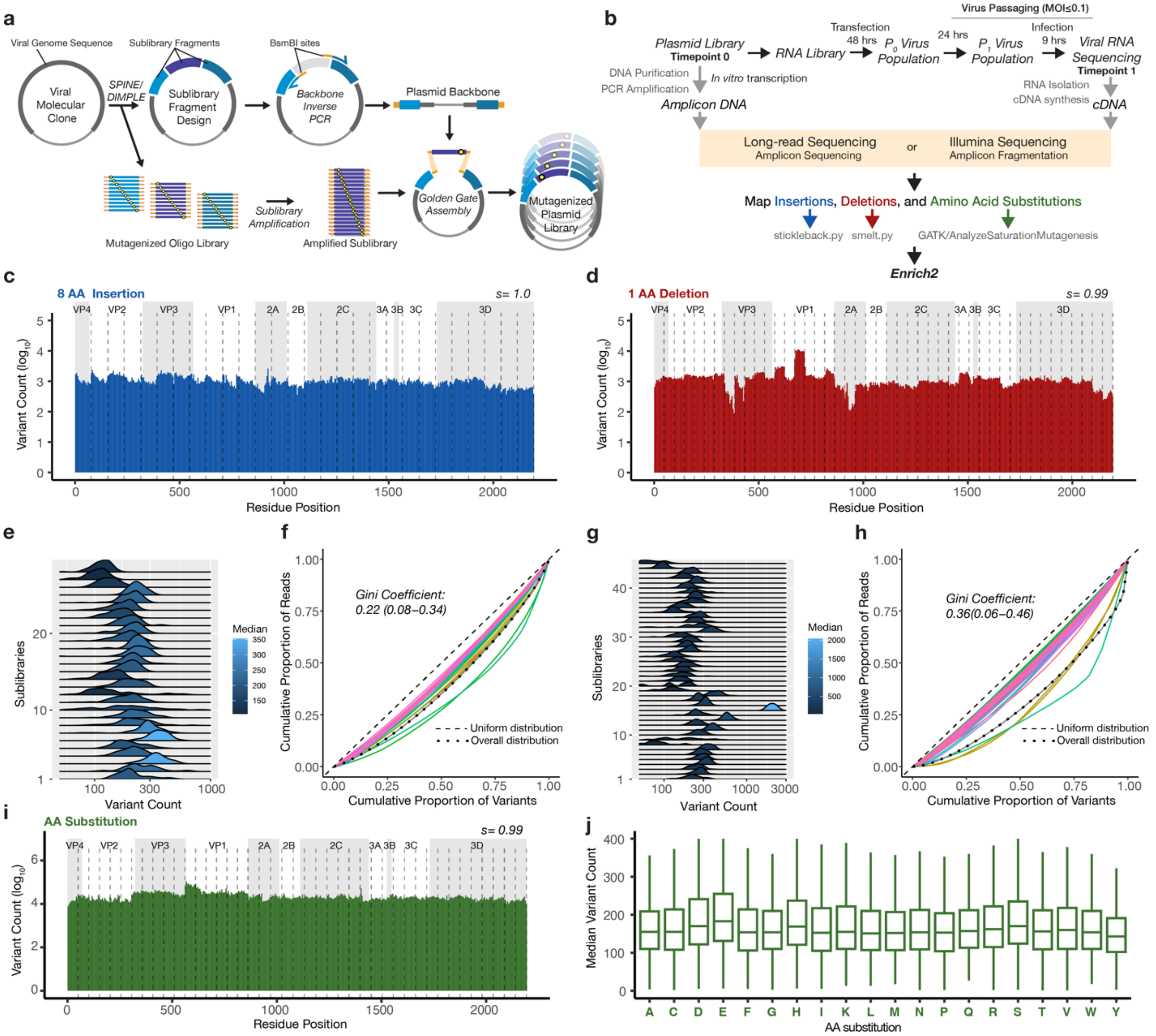

Supplementary Figure 1. Deep Insertional, Deletional, and Mutational Scanning of the EV-A71 proteome (a) Schematic overview of the steps for generating Deep Mutational Scanning libraries, including library design through SPINE or DIMPLE and the cloning steps for library construction. (b) Workflow of the experimental virology pipeline to rescue virus from plasmid libraries and the subsequent sequencing steps at different timepoints to measure variant frequency change in the population. The technologies used for sequencing and the analysis pipelines to detect different variants are detailed. Bar plot (bin=5) showing the variant counts across the viral proteome for insertions (c), deletions (d), and AA substitutions (i). The distribution of variants is shown through ridge kernel line plots and Lorenz curves for insertions (e,f) and deletions (g,h). (j) Box plot showing the median counts of the different AA substitution variants. The lower hinge of the box plot represents the 25th percentiles and the upper hinge represents the 75th percentiles, with the center line showing the median. The whiskers extend to the smallest and largest values within 1.5 times the interquartile range (IQR) from the hinges. The saturation (s) is defined as the proportion of detected variants (with a count higher than one) to the total number of designed variants.

EV-A71 Deep Mutation, Insertion, and Deletion Scanning
Bakhache, et al.
Nature Microbiology
Supplementary Information

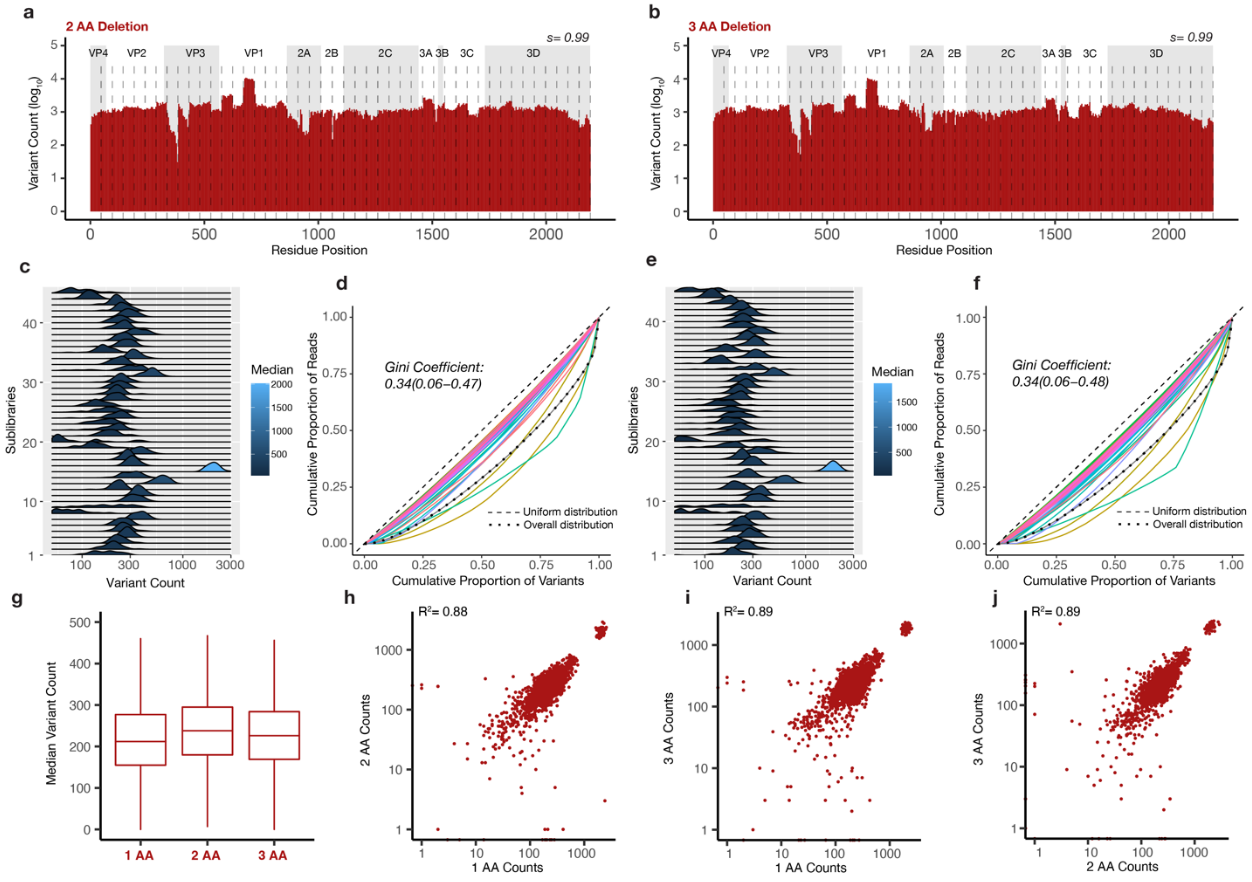

Supplementary Figure 2. Deep Deletion Scanning of the EV-A71 proteome Barplot (bin=5) showing the variant counts across the viral proteome for 2 AA (a) and 3 AA (b) deletions. The distribution of variants is shown through ridge kernel line plots and Lorenz curves for (c-d) 2 AA deletions and (e-f) 3 AA deletions. (g) Boxplot showing the median variant counts for different deletion lengths. The lower hinge of the box plot represents the 25th percentiles and the upper hinge represents the 75th percentiles, with the center line showing the median. The whiskers extend to the smallest and largest values within 1.5 times the interquartile range (IQR) from the hinges. Scatter plots comparing variant counts for (h) 1 AA and 2 AA deletions, and (i) 1 and 3 AA deletions, and (j) 2 and 3 AA deletions.

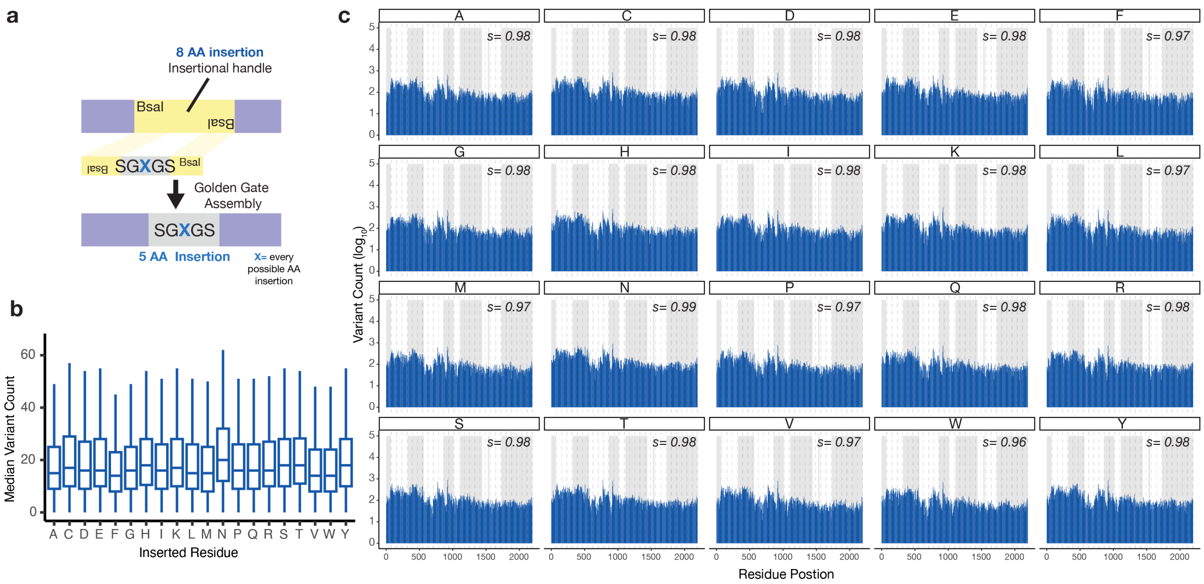

Supplementary Figure 3. Deep Insertion Scanning of the EV-A71 proteome (a) Schematic presentation of the insertional handle used to generate a 5 AA insertion library, where each inserted sequence consists of one of the 20 AAs flanked by flexible linkers. (b) Boxplot showing the median variant counts for the different inserted residues. The lower hinge of the box plot represents the 25th percentiles and the upper hinge represents the 75th percentiles, with the center line showing the median. The whiskers extend to the smallest and largest values within 1.5 times the interquartile range (IQR) from the hinges. (c) Barplots (bin=5) displaying the variant counts for all inserted residues across the viral proteome.
