# Supplementary material for: Deep mutation, insertion and deletion scanning across the Enterovirus A proteome reveals constraints shaping viral evolution
Source: Nat Microbiol. 2024 Nov 28;10(1):158–68. doi: 10.1038/s41564-024-01871-y (PMC11726453; doi:10.1038/s41564-024-01871-y)
Supplement: Supplementary file 2 — Reporting Summary [file 41564_2024_1871_MOESM2_ESM.pdf]

nature portfolio

Corresponding author(s): Patrick T. Dolan

Last updated by author(s): Oct 10, 2024

Reporting Summary

Nature Portfolio wishes to improve the reproducibility of the work that we publish. This form provides structure for consistency and transparency in reporting. For further information on Nature Portfolio policies, see our Editorial Policies and the Editorial Policy Checklist.

Statistics

For all statistical analyses, confirm that the following items are present in the figure legend, table legend, main text, or Methods section.

n/a	Confirmed	
☐	☒	The exact sample size (*n*) for each experimental group/condition, given as a discrete number and unit of measurement
☐	☒	A statement on whether measurements were taken from distinct samples or whether the same sample was measured repeatedly
☐	☒	The statistical test(s) used AND whether they are one- or two-sided<br>*Only common tests should be described solely by name; describe more complex techniques in the Methods section.*
☒	☐	A description of all covariates tested
☐	☒	A description of any assumptions or corrections, such as tests of normality and adjustment for multiple comparisons
☐	☒	A full description of the statistical parameters including central tendency (e.g. means) or other basic estimates (e.g. regression coefficient) AND variation (e.g. standard deviation) or associated estimates of uncertainty (e.g. confidence intervals)
☐	☒	For null hypothesis testing, the test statistic (e.g. *F*, *t*, *r*) with confidence intervals, effect sizes, degrees of freedom and *P* value noted<br>*Give P values as exact values whenever suitable.*
☒	☐	For Bayesian analysis, information on the choice of priors and Markov chain Monte Carlo settings
☒	☐	For hierarchical and complex designs, identification of the appropriate level for tests and full reporting of outcomes
☒	☐	Estimates of effect sizes (e.g. Cohen's *d*, Pearson's *r*), indicating how they were calculated

Our web collection on statistics for biologists contains articles on many of the points above.

Software and code

Policy information about availability of computer code

Data collection	To collect sequencing data and analyse, we used both commercial sequencing software (illumina bcl2fastq/2.20, guppy_gpu/6.0.6 or guppy/6.5.7. and minimap2/2.24 or minimap2/2.26) for mapping insertions or deletions, we used either stickleback (insertions), or deletionmapper0.2.py (deletions). These were both developed in house to analyse these data. These scripts are included in the repository included in the manuscript https://doi.org/10.5061/dryad.866t1g1xm.
Data analysis	Python scripts used in mapping the reads with engineered insertions and deletions are available at https://github.com/QVEU/InDel_Toolkit. The R scripts associated with specific bioinformatic computations performed herein are available at https://github.com/QVEU/eva71_dimple. Rscripts to regenerate all the figures are included in the dryad repository associated with this manuscript, along with all the python and R script versions used for this study: https://doi.org/10.5061/dryad.866t1g1xm.<br><br>R scripts for analysis and figure generation were run using the R version 4.0.3 (2020-10-10). R packages used were: ggplot2, tidyverse, tidyr, ggpubr, dplyr, ggridges, ineq, RColorBrewer, stringr, gglorenz, readr, scales, zoo, Biostrings, DescTools. AnalyzeSaturationMutagenesis in GATK version 4.2.6.0 or 4.5.0.0 was used for analysis of amino acid change experiments. The Enrich2 software (v1.3.1) was used for calculation of enrichment scores. Chimera sessions were created in Chimera production version 1.16 (build 42360).

For manuscripts utilizing custom algorithms or software that are central to the research but not yet described in published literature, software must be made available to editors and reviewers. We strongly encourage code deposition in a community repository (e.g. GitHub). See the Nature Portfolio guidelines for submitting code & software for further information.

Data

Policy information about availability of data

All manuscripts must include a data availability statement. This statement should provide the following information, where applicable:

- Accession codes, unique identifiers, or web links for publicly available datasets
- A description of any restrictions on data availability
- For clinical datasets or third party data, please ensure that the statement adheres to our policy

All raw sequencing read data is available in the SRA database under NCBI project number, PRJNA1066851. Structures used in this manuscript are available through these accession codes: PDB: 3W95 (EV-A71 2A), PDB: 5GQ1 (EV-A71 2C), PDB: 6HLW (EV-A71 3A), PDB: 3OSY (EV-A71 3C), PDB: 3N6L or 6KWQ (EV-A71 3D), PDB: 8E2X (EV-A71 virion), and PDB: 7QW9 (CV-A6 virion).

Research involving human participants, their data, or biological material

Policy information about studies with human participants or human data. See also policy information about sex, gender (identity/presentation), and sexual orientation and race, ethnicity and racism.

Reporting on sex and gender	NA
Reporting on race, ethnicity, or other socially relevant groupings	NA
Population characteristics	NA
Recruitment	NA
Ethics oversight	NA

Note that full information on the approval of the study protocol must also be provided in the manuscript.

Field-specific reporting

Please select the one below that is the best fit for your research. If you are not sure, read the appropriate sections before making your selection.

☒ Life sciences ☐ Behavioural & social sciences ☐ Ecological, evolutionary & environmental sciences

For a reference copy of the document with all sections, see nature.com/documents/nr-reporting-summary-flat.pdf

Life sciences study design

All studies must disclose on these points even when the disclosure is negative.

Sample size	Sample size was determined based on previous studies using deep-mutational scanning in Enteroviruses, and the depth and complexity of the libraries. Based on previous studies three biological replicates was sufficient to distinguish strong fitness effects. The studies used as a reference were: https://elifesciences.org/articles/64256 and https://journals.plos.org/plosbiology/article?id=10.1371/journal.pbio.3002709
Data exclusions	No data were excluded from the analyses.
Replication	Data represent three biological replicates of each screen. Replication attempts were successful and showed high correlation.
Randomization	Randomization is not relevant in these studies. However, related to this idea, these studies involve large pools of mutants maintained at population sizes that ensure well-balanced representation (and verified by sequencing). Care is taken, through propagation to mitigate any bottlenecking which might bias results. These large screens are performed in biological replicate to identify sampling bias or error in replicate populations.
Blinding	No blinding was used or appropriate for this study. We deal with high-throughput sequencing data that includes analysis using standardized data analysis frameworks. Knowledge of samples does not affect outcome of the analysis.

Reporting for specific materials, systems and methods

We require information from authors about some types of materials, experimental systems and methods used in many studies. Here, indicate whether each material, system or method listed is relevant to your study. If you are not sure if a list item applies to your research, read the appropriate section before selecting a response.

Materials & experimental systems

n/a	Involved in the study
☒	☐ Antibodies
☐	☒ Eukaryotic cell lines
☒	☐ Palaeontology and archaeology
☒	☐ Animals and other organisms
☒	☐ Clinical data
☒	☐ Dual use research of concern
☒	☐ Plants

Methods

n/a	Involved in the study
☒	☐ ChIP-seq
☒	☐ Flow cytometry
☒	☐ MRI-based neuroimaging

Eukaryotic cell lines

Policy information about cell lines and Sex and Gender in Research

Cell line source(s)	Source: American Type Culture Collection (ATCC). Cell line used is Rhabdomyosarcoma (RD) (ATCC, CCL-136)
Authentication	Cells have been characterized by genotyping, confirming them as Rhabdomyosarcoma cells.
Mycoplasma contamination	All cells tested negative for mycoplasma contamination by isothermal amplification prior to the experiment.
Commonly misidentified lines (See ICLAC register)	Cell lines are not listed in the database. Cell lines verified genetically as RD, rhabdomyosarcoma. Tested cells were from lot and passage of those used in this study.

Plants

Seed stocks	*Report on the source of all seed stocks or other plant material used. If applicable, state the seed stock centre and catalogue number. If plant specimens were collected from the field, describe the collection location, date and sampling procedures.*
Novel plant genotypes	*Describe the methods by which all novel plant genotypes were produced. This includes those generated by transgenic approaches, gene editing, chemical/radiation-based mutagenesis and hybridization. For transgenic lines, describe the transformation method, the number of independent lines analyzed and the generation upon which experiments were performed. For gene-edited lines, describe the editor used, the endogenous sequence targeted for editing, the targeting guide RNA sequence (if applicable) and how the editor was applied.*
Authentication	*Describe any authentication procedures for each seed stock used or novel genotype generated. Describe any experiments used to assess the effect of a mutation and, where applicable, how potential secondary effects (e.g. second site T-DNA insertions, mosiacism, off-target gene editing) were examined.*
